# Effect of Hydrogen Peroxide on Cyanobacterial Biofilms

**DOI:** 10.3390/antibiotics12091450

**Published:** 2023-09-16

**Authors:** Maria João Romeu, João Morais, Vítor Vasconcelos, Filipe Mergulhão

**Affiliations:** 1 LEPABE—Laboratory for Process Engineering, Environment, Biotechnology and Energy, Faculty of Engineering, University of Porto, Rua Dr. Roberto Frias, 4200-465 Porto, Portugal; mariaromeu@fe.up.pt; 2 ALiCE—Associate Laboratory in Chemical Engineering, Faculty of Engineering, University of Porto, Rua Dr. Roberto Frias, 4200-465 Porto, Portugal; 3 CIIMAR—Interdisciplinary Centre of Marine and Environmental Research, University of Porto, Terminal de Cruzeiros do Porto de Leixões, Av. General Norton de Matos s/n, 4450-208 Matosinhos, Portugal; jmorais@ciimar.up.pt (J.M.); vmvascon@fc.up.pt (V.V.); 4 Department of Biology, Faculty of Sciences, University of Porto, Rua do Campo Alegre, 4169-007 Porto, Portugal

**Keywords:** cyanobacterial biofilms, marine biofouling, antifouling strategies, chemical disinfection, hydrogen peroxide, resistance, virulence

## Abstract

Although a range of disinfecting formulations is commercially available, hydrogen peroxide is one of the safest chemical agents used for disinfection in aquatic environments. However, its effect on cyanobacterial biofilms is poorly investigated. In this work, biofilm formation by two filamentous cyanobacterial strains was evaluated over seven weeks on two surfaces commonly used in marine environments: glass and silicone-based paint (Sil-Ref) under controlled hydrodynamic conditions. After seven weeks, the biofilms were treated with a solution of hydrogen peroxide (H_2_O_2_) to assess if disinfection could affect long-term biofilm development. The cyanobacterial biofilms appeared to be tolerant to H_2_O_2_ treatment, and two weeks after treatment, the biofilms that developed on glass by one of the strains presented higher biomass amounts than the untreated biofilms. This result emphasizes the need to correctly evaluate the efficiency of disinfection in cyanobacterial biofilms, including assessing the possible consequences of inefficient disinfection on the regrowth of these biofilms.

## 1. Introduction

Marine biofouling remains a global concern. Numerous approaches have been used to prevent, delay, and control the formation of marine biofilms and the establishment of fouling communities, including the development of antifouling coatings, the use of cleaning technologies and disinfection procedures, and the application of additional methodologies, such as hydrodynamic/acoustic cavitation and thermal stress [1]. The availability of disinfection treatments to mitigate the effects of marine biofouling is an important requirement for effectively managing marine biosecurity risks, as well as alleviating the consequences of biofouling, including the impact of contamination and/or corrosion, economic costs, and ecological consequences [2,3,4,5].

Various aquatic disinfectants have been used as a control treatment for cleaning and disinfecting marine equipment, such as NALCO^®^ 79,125 Safe Acid (sulfamic acid: 60–100%), Rydlyme^®^ (hydrogen chloride: <10%), Triple7 Enviroscale Plus^®^ (citric acid: 30–60%; lactic acid: 30–60%), TermoRens^®^ Liquid 104 cleansing fluid (5–15% citric acid and <10% phosphoric acid), Barnacle Buster^®^ (85% phosphoric acid), and Descalex^®^ (sulfamic acid: 60–100%) [6,7,8,9]. These can be used in liquid, powder, and/or tablet form and can be applied through spraying or immersing equipment in a disinfectant solution. It is of utmost importance that the disinfection agent is safe, non-toxic, and biodegradable in marine environments. Virkon^®^ Aquatic, a widely known agent in the peroxygen family, is 99.9% biodegradable and breaks down to water and oxygen [10]. Additionally, it is listed among the disinfectants registered by the U.S. Environmental Protection Agency, designed particularly for application within aquaculture facilities to combat aquatic bacterial, fungal, and viral pathogens [11,12].

Oxidative stress-based treatments are also a major approach to prevent or mitigate cyanobacterial blooms [13,14,15,16]. Oxidizing agents, including hydrogen peroxide (H_2_O_2_), are broad-spectrum disinfectant agents and their costs are relatively low [9]. Although the catalase produced by bacteria may protect cells from H_2_O_2_ disinfection, this defense is overwhelmed by the concentrations used during chemical treatment (generally, a 5–20% H_2_O_2_ solution is bactericidal, fungicidal, and virucidal) [17]. Reactive hydroxyl radicals produced by H_2_O_2_ cause the oxidation of lipids, proteins, and DNA, leading to severe oxidative stress. One notable benefit associated with the use of H_2_O_2_ is related to its degradation to water and oxygen within a few days, thus, it does not persist in marine environments [18]. Notably, some studies reported that the use of H_2_O_2_ to suppress cyanobacterial blooms can promote a short-term pulse disturbance in the ecosystem but is not detrimental to microbial communities [15]. Additional studies reported that H_2_O_2_ may affect other aquatic organisms [19,20]. The reported dose–response observations for H_2_O_2_ on cyanobacteria vary extensively from 1 to 100 mg/L [8,15,16,20,21,22,23]. However, all these studies were performed on planktonic cyanobacteria, and there are few works focused on the impact of H_2_O_2_ disinfection treatments on marine cyanobacterial biofilms. Moreover, most of these studies focused on the microcystin-producing cyanobacterium *Microcystis* [8,23,24], and few of them were performed on filamentous cyanobacteria [22]. 

Since marine biofouling includes the initial formation of biofilms by marine bacteria and diatoms (microfouling), followed by the establishment of larger fouling communities on submerged devices and equipment (macrofouling), a promising approach for biofouling mitigation is to manage macrofouling by delaying microfouling events [1]. Since cyanobacteria are responsible for starting biofilm formation on submerged surfaces, constitute the major components of marine biofilms [25,26,27], and excrete large amounts of EPS [28,29], which are relevant for biofilm cohesion, stability, and structure [30], these microorganisms may determine the function and arrangement of biofilms [31,32]. Moreover, it has also been considered that cyanobacteria can establish symbiotic relationships with diatoms in marine biofilms [33,34], and biofilm formation by bacteria and algae is critical to the successive attachment of macrofouler organisms [26,35]. Therefore, cyanobacterial strains were used to evaluate the impact of H_2_O_2_ disinfection in the present work.

In this context, the aim of the current work was to evaluate the biofilm-forming ability of two cyanobacterial strains on two different surfaces used in marine environments (glass and a commercial marine coating, a foul-release silicone-based paint (Sil-Ref)) and characterize the impact of H_2_O_2_ disinfection on these biofilms. The impact of the treatment on the further development of these biofilms was also assessed to investigate longer-term effects.

## 2. Results

### 2.1. Biofilm Development

**Figure 1:**
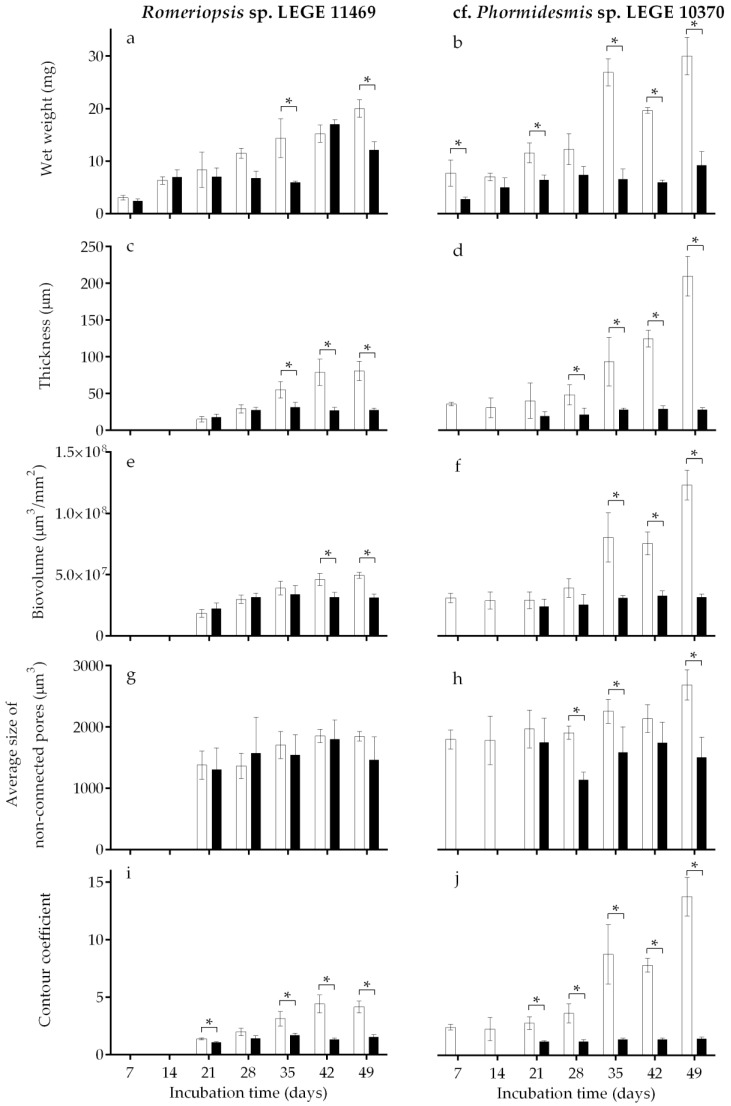
*Romeriopsis* sp. LEGE 11469 and cf. *Phormidesmis* sp. LEGE 10370 biofilm development on different surfaces (glass—white bars and Sil-Ref—black bars). The parameters analyzed refer to biofilm wet weight (**a**,**b**), thickness (**c**,**d**), biovolume (**e**,**f**), average size of non-connected pores (**g**,**h**), and contour coefficient (**i**,**j**). Mean values and SD from two biological assays with two technical replicates each are represented. For each sampling day, the asterisk (*) indicates significant differences between surfaces (*p* ≤ 0.1; unpaired, non-parametric Mann–Whitney test).

The biofilm-forming ability of both cyanobacterial strains on two different surfaces (glass and Sil-Ref) was assessed over 7 weeks (49 days). The results obtained for the biofilm wet weight, thickness, biovolume, average size of non-connected pores, and contour coefficient are presented in Figure 1, but for all these parameters (except the wet weight), the results are only shown from day 21 onwards. This is because in the first two sampling weeks, biofilm development was below the detection limit of OCT.

In general, a gradual temporal increase in biofilm wet weight (Figure 1a,b), thickness (Figure 1c,d), and biovolume (Figure 1e,f) were observed, proving that both cyanobacterial strains are good biofilm formers. However, this biofilm development was more evident on glass than on the Sil-Ref surface for both cyanobacterial strains. In fact, for *Romeriopsis* sp. LEGE 11469, increases of 85% and 80% in the biofilm wet weight were observed from day 7 to day 49, and increases of 81% and 35% in the biofilm thickness and 63% and 29% in the biovolume were observed from day 21 to day 49 on the glass and Sil-Ref, respectively. Likewise, for cf. *Phormidesmis* sp. LEGE 10370, increases of 74% and 69% in the biofilm wet weight were observed from day 7 to day 49, and increases of 81% and 31% in the biofilm thickness and 76% and 24% in the biovolume were observed from day 21 to day 49 on the glass and Sil-Ref, respectively. Moreover, biofilm development was lower on the Sil-Ref surface compared to the glass (Figure 1a–f). Overall, the major differences observed in biofilm development on the glass and Sil-Ref were observed from day 35, regardless of the cyanobacterial strain. Indeed, the biofilm mass, thickness and biovolume obtained on the Sil-Ref were on average 25%, 36%, and 16% lower, respectively, when compared to the values obtained on the glass for *Romeriopsis* sp. LEGE 11469 and on average 56%, 68%, and 49% lower, respectively, when compared to the values obtained on the glass for cf. *Phormidesmis* sp. LEGE 10370.

Regarding the quantitative values of the biofilm structure obtained from 3D OCT analysis, no statistical differences were observed between the surfaces regarding the average size of non-connected pores inside the biofilm structure for *Romeriopsis* sp. LEGE 11469 (Figure 1g). In turn, for cf. *Phormidesmis* sp. LEGE 10370, a higher average size of these non-connected pores was observed for biofilms developed on the glass (Figure 1h). The contour coefficient parameter facilitates the assessment of the biofilm fraction that interfaces with its surrounding environment. [36]. Consequently, values nearing 1 indicate a flat and homogeneous biofilm, whereas values exceeding 1 correspond to biofilms with heterogeneous structures, such as those containing streamers. The results obtained from both cyanobacterial strains indicated that the biofilms formed on the Sil-Ref presented a more homogeneous structure, while the biofilms that formed on the glass were more heterogeneous and this heterogeneity generally increased over time (Figure 1i,j).

When comparing the biofilm-forming ability of both cyanobacterial strains, cf. *Phormidesmis* sp. LEGE 10370 seems to be a better biofilm former, comparing the values of the biofilm wet weight, thickness, and biovolume reached on glass (Figure 1a–f, white bars). In turn, slight differences were observed for biofilms formed on the Sil-Ref by both cyanobacterial strains (Figure 1a–f, black bars).

### 2.2. H_2_O_2_ Disinfection Treatment and Cyanobacterial Biofilm Regrowth

**Figure 2:**
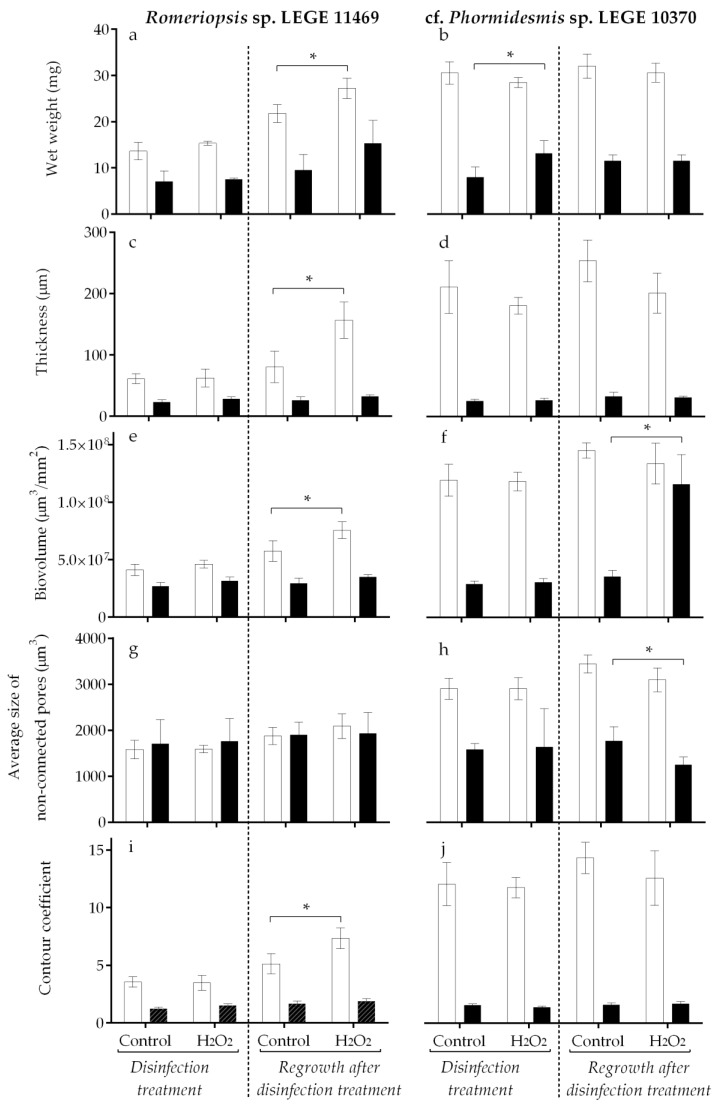
*Romeriopsis* sp. LEGE 11469 and cf. *Phormidesmis* sp. LEGE 10370 biofilm development on different surfaces (glass—white bars and Sil-Ref—black bars) after H_2_O_2_ disinfection treatment (left side) and regrowth for two weeks after the H_2_O_2_ disinfection treatment (right side). The parameters analyzed refer to biofilm wet weight (**a**,**b**), thickness (**c**,**d**), biovolume (**e**,**f**), average size of non-connected pores (**g**,**h**), and contour coefficient (**i**,**j**). Mean values and SD from two biological assays with two technical replicates each are represented. For each comparison Control vs. H_2_O_2_ treatment, the asterisk (*) indicates significant differences between conditions (*p* ≤ 0.1; unpaired, non-parametric Mann–Whitney test).

After 7 weeks of biofilm development, the susceptibility of cyanobacterial biofilms to disinfection treatment was evaluated by applying 100 mg/L H_2_O_2_ to the biofilms already formed. After 4 h of treatment, the biofilms were analyzed regarding their biofilm wet weight, thickness, biovolume, average size of non-connected pores, and contour coefficient. The results obtained after H_2_O_2_ application for both cyanobacterial strains and both surfaces are presented in Figure 2 (left side). To evaluate the effect of this chemical procedure on long-term cyanobacterial biofilm development, the biofilms previously subjected to H_2_O_2_ treatment were allowed to grow for an additional two weeks. After this time, the biofilms were analyzed again, and the results are represented in Figure 2 (right side).

The results obtained immediately after the 4 h H_2_O_2_ disinfection (Figure 2, left side) indicated that both cyanobacterial biofilms presented higher tolerance to chemical disinfection. Indeed, for *Romeriopsis* sp. LEGE 11469, and for both surfaces, no statistical differences were observed between the control and the biofilms subjected to H_2_O_2_ disinfection (Figure 2a,c,e,g,i, left side). Likewise, for cf. *Phormidesmis* sp. LEGE 10370, only a slight increase in the biofilm wet weight was observed on biofilms formed on the Sil-Ref after the four hours of H_2_O_2_ disinfection (Figure 2b, *p* = 0.057).

The results achieved for biofilm development after chemical disinfection (regrowth for 2 weeks) demonstrated larger variations (Figure 2, right side) and mainly on biofilms formed by *Romeriopsis* sp. LEGE 11469 on glass (Figure 2a,c,e,i, right side). Indeed, two weeks after the treatment, these biofilms showed an increase in the biofilm wet weight (*p* = 0.057), biofilm thickness (*p* = 0.003), biovolume (*p* = 0.002), and contour coefficient (*p* = 0.001) when compared to the biofilms not exposed to chemical treatment (control). Notably, on the biofilms formed by cf. *Phormidesmis* sp. LEGE 10370, differences after the chemical treatment were observed only for biofilms formed on the Sil-Ref (Figure 2f,h, right side). These biofilms showed an increase in the biovolume (*p* = 0.004) and a decrease in the average size of the non-connected pores (*p* = 0.003).

**Figure 3:**
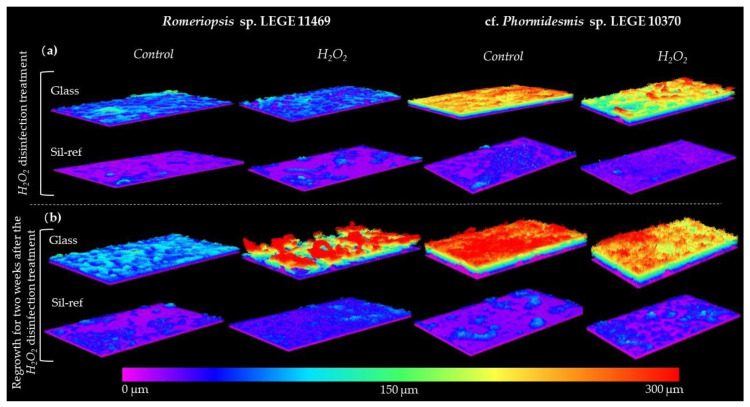
Representative 3D OCT images of *Romeriopsis* sp. LEGE 11469 and cf. *Phormidesmis* sp. LEGE 10370 biofilms formed on glass and Sil-Ref after (**a**) H_2_O_2_ disinfection treatment and (**b**) regrowth for two weeks after the H_2_O_2_ disinfection treatment. The color scale shows the biofilm thickness (µm).

Figure 3 shows representative 3D OCT images of cyanobacterial biofilms on both surfaces immediately after the H_2_O_2_ disinfection treatment (a) and two weeks after treatment (b). All the images corroborate the quantitative data on biofilm biomass, thickness, and structure, which were previously represented in Figure 2. A similar biofilm structure was obtained between the non-treated and treated samples immediately after chemical disinfection (Figure 3a). Major differences can be observed in the images obtained after the two weeks of regrowth (Figure 3b). The increase in the biofilm thickness, biovolume and a more heterogeneous structure was observed on the glass in biofilms formed by *Romeriopsis* sp. LEGE 11469 (Figure 3b). The higher biofilm thickness (around 300 µm) and biovolume reached by the biofilms formed by cf. *Phormidesmis* sp. LEGE 10370 on glass, as well as the higher heterogeneous architecture of these biofilms, can also be confirmed on these representative images (Figure 3b).

## 3. Discussion

In the first stage of this work, the biofilm-forming ability of two different filamentous cyanobacteria strains on two different marine surface materials (glass and Sil-Ref) was evaluated for 49 days under controlled hydrodynamic conditions, which mimic real marine environments.

Overall, cyanobacterial biofilm development was lower on the Sil-Ref surface compared to glass. From the results obtained in the current work, it is also possible to infer that Sil-Ref surfaces may promote the development of a biofilm with homogeneous architecture. Previous studies performed with coccoid cyanobacteria also demonstrated the potential of Sil-Ref in inhibiting cyanobacterial biofilm formation [37,38] by decreasing the number of biofilm cells, chlorophyll *a* content, and biofilm thickness compared to other tested surfaces [38]. Moreover, as observed in the present study, Sil-Ref surfaces showed high efficacy in reducing biofilm formation during the maturation stage of biofilm development (from day 35 onwards) [37]. The Sil-Ref surface can prevent hydrophobic or hydrophilic interactions by reducing the adsorption of proteins and bacteria since this coating combines hydrogel polymers with silicone fouling-release technology to originate polydimethylsiloxane matrices with self-stratifying hydrogel-promoting polymers [39]. Among the biocide-free coating technologies, fouling-release coatings are the most intensively used in the marine industry due to their eco-friendly biocide-free antifouling properties, acting by physical–chemical and mechanical mechanisms, showing long-term efficiency, particularly in high dynamic systems [40]. Moreover, silicone polymers have been commonly used in fouling-release coatings due to their elasticity, heat resistance, and effectiveness against the adhesion and attachment of common marine algae, diatoms, barnacles, and mussels [41].

Although both *Romeriopsis* sp. LEGE 11469 and cf. *Phormidesmis* sp. LEGE 10370 strains can be considered good biofilm formers, the cyanobacterium cf. *Phormidesmis* sp. LEGE 10370 revealed a higher potential to form a robust biofilm, reaching higher values of biofilm thickness, biovolume, and wet weight over the seven weeks. Indeed, previous studies on filamentous cyanobacteria showed that biofilm-forming ability could be related to specific strain morphology and also its site of isolation [36,38,42].

The susceptibility of cyanobacterial biofilms to H_2_O_2_ disinfection treatment was evaluated after 7 weeks of biofilm development. The results obtained in the present study indicated that both cyanobacterial biofilms presented higher tolerance to the chemical treatment. Although the use of H_2_O_2_ disinfection has been evaluated and used effectively to control cyanobacterial blooms [8,20,43] and the control of invasive species, such as the killer shrimp, *Dikerogammarus villosus* [6], it seems that this approach is not suitable to reduce mature cyanobacterial biofilms, at least biofilms formed by these cyanobacterial strains and by the time–concentration of H_2_O_2_ used. For example, while at a concentration of 10 mg/L, maximal cell death was observed for the unicellular cyanobacterium *Microcystis* [8]. In addition, Petrille and Miller [44] reported a 90% mortality rate of adult zebra mussels, *Dressena polymorpha*, after exposure to 5.4 mg/L for 21 days. At higher concentrations of 10, 20, and 40 mg/L, 100% mortality was attained after 8, 9, and 3 days, respectively. The Asian clam, *Corbicula fluminea*, was shown to be more tolerant to H_2_O_2_ at the same tested concentrations, with 100% mortality achieved after 14, 10, and 9 days, respectively [44]. An additional study investigated the dual effect of H_2_O_2_ application for oxygen enrichment and disinfection when continuously applied to a recirculating aquaculture system for rearing European seabass [45]. H_2_O_2_ addition, equivalent to 2.4 and 15.8 mg/L of H_2_O_2_, was applied for 4 h per day in three 5-day experiments. The results showed that 15.8 mg/L of H_2_O_2_ increased the oxygen levels in the tank water while reducing the microbial load and did not affect the fish. In another study aimed at preventing the spread of highly invasive killer shrimp, it was shown that concentrations from 10.000 to 40.000 mg/L of H_2_O_2_ may limit its spread [6].

It is well known that in biofilms, organisms become more tolerant to several antimicrobial agents and disinfection methods used in marine environments [46], and the lower susceptibility of these biofilms to this oxidizing agent may be related to the neutralization of this compound by organic matter found in the biofilm matrix. In the specific case of H_2_O_2,_ its sensitivity is deeply affected by several factors, including light [47], nutrient level [23], cell density, and the stage of cell growth [48]. Likewise, maintaining effective treatment concentrations presents a logistical challenge due to the rapid degradation of H_2_O_2_ in seawater. Therefore, limitations of the effectiveness of disinfectants on marine biofilm mitigation can be related to several issues. First, a lack of comprehensive data hinders the accurate assessment of efficiency against all pertinent biofouling groups. Subsequently, most chemical compound concentrations require vigilant monitoring due to their efficacy being contingent upon various factors, and this monitoring is logistically and technically challenging [6,9,11,12,49]. In addition, the absence of mechanical/cleaning treatment prior to disinfection can explain the high tolerance obtained in the present study. For example, the mitigation of internal corrosion in petroleum pipelines includes mechanical cleaning and the application of a disinfectant, which is applied in batch mode (once a week for a few hours) or continuously injected. However, disinfectants alone do not provide adequate mitigation of internal corrosion control since the compound cannot penetrate deeply enough into thick biofilms [50,51].

Although H_2_O_2_ treatment showed no differences in the cyanobacterial biofilms immediately after application, this chemical procedure may impact long-term cyanobacterial biofilm development. After continuous biofilm growth for two additional weeks after the treatment, the biofilms developed on glass by *Romeriopsis* sp. LEGE 11469 showed an increase in the wet weight, thickness, and biovolume compared to the untreated biofilms. However, this behavior was strain and surface-dependent, since for biofilms formed by cf. *Phormidesmis* sp. LEGE 10370, differences after the chemical treatment were observed only for biofilms formed on the Sil-Ref, which showed an increase in the biovolume and a decrease in the average size of the non-connected pores. However, both findings demonstrated that cyanobacterial biofilms exposed to disinfectants can display an enhanced biofilm-forming ability. Few studies have focused on the impact of H_2_O_2_ disinfection after a long time of exposure in cyanobacteria [21,52,53]. Moreover, all of them evaluated the effect on planktonic bacteria and/or cyanobacterial blooms. For example, Matthijs and co-workers showed that after H_2_O_2_ application and the subsequent cyanobacterial collapse, the concentration of cyanobacteria in a lake remained low for seven weeks [53]. In addition, Sinha and co-workers observed that the longevity of 2.5 and 4.0 mg/L H_2_O_2_ treatment effects lasted for up to 5 weeks [52]. However, previous studies on biofilms with different bacteria have already shown that oxidizing agents, including H_2_O_2_, may contribute to enhanced biofilm development [54,55]. This concern holds significance as it implies that employing disinfecting agents at sub-lethal levels may enhance the bacterial capacity to form biofilms and amplify their presence in marine environments.

Reevaluating the utilization of H_2_O_2_ as a disinfectant is recommended, particularly in relation to the disinfection of submerged marine surfaces prone to biofilm colonization. This is crucial to prevent the onset of resistance resulting from exposure to sub-lethal concentrations. Overall, it is critical to assess and understand the phenotypic characteristics of biofilms after exposure to chemical treatments since this information can help us understand the mechanisms involved in resistance and develop more efficient alternative treatments.

## 4. Materials and Methods

**Figure 4:**
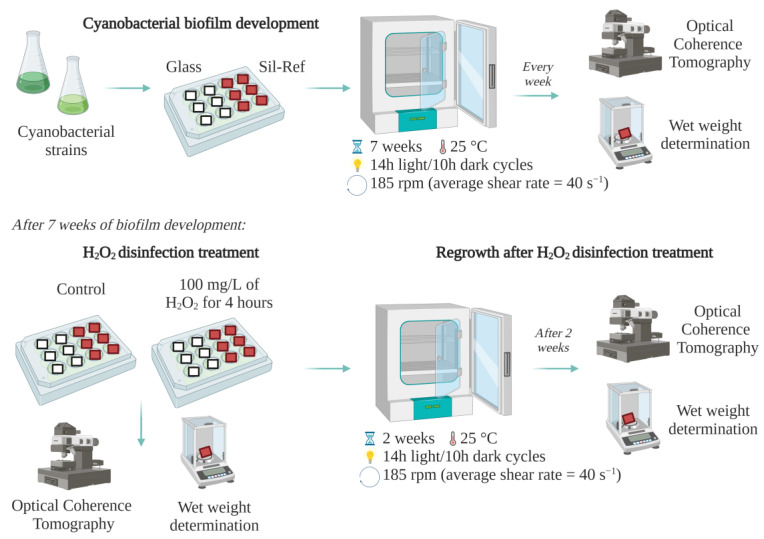
Diagram of the experimental stages of the current study.

The flowchart in Figure 4 illustrates the experimental procedures detailed in the following sections.

### 4.1. Organism and Culture Conditions

Two different cyanobacterial strains were used (*Romeriopsis* sp. LEGE 11469 and cf. *Phormidesmis* sp. LEGE 10370). These were obtained from the Blue Biotechnology and Ecotoxicology Culture Collection (LEGE-CC) from CIIMAR, Portugal [56,57]. *Romeriopsis* sp. LEGE 11469 was isolated from a subtidal sample, epilithic (13m depth), about 200 m off the shore near Castelo do Queijo, Portugal (41.185809 N 8.719079 W). In turn, cf. *Phormidesmis* sp. LEGE 10370 was isolated from an intertidal zone, scraped from a marine sponge at Praia da Memória, Portugal (41.23119 N 8.721750 W). Cells from a starting culture were cultivated for 30 days in a 750 mL culture using Z8 medium [58] enriched with 25 g/L of synthetic sea salts (Tropic Marin) and vitamin B12 (Sigma Aldrich, Merck, Saint Louis, MO, USA). These cultures were maintained with a light cycle of 14 h (10–30 μmol photons m^−2^ s^−1^) followed by 10 h of darkness, at a temperature of 25 °C.

### 4.2. Biofilm Formation

The biofilms were developed on glass (Vidraria Lousada, Lda, Portugal) coupons (1 cm^2^) and on a commercial marine coating (foul-release silicone-based paint (Sil-Ref, HEMPASIL X3+ 87500, Copenhagen, Denmark) coupons (1 cm^2^)). Glass is a prevalent artificial submerged surface encountered on various types of equipment within aquatic and marine settings, including underwater windows of vessels, aquaculture apparatus, flotation spheres, moored buoys, and underwater cameras as well as measuring instruments or sensors [59,60,61]. The hydrogel-based fouling-release coating formulated with silicone represents a third-generation coating commonly applied to grids, water inlet piping, and ship hulls [37]. Surface sterilization and preparation were performed as previously described [37,42]. Briefly, glass coupons were sterilized through immersion in a 2% (*v*/*v*) TEGO 2000^®^ solution (JohnsonDiversey, Northampton, UK) for 20 min under agitation (150 rpm). They were subsequently rinsed with sterile distilled water to eliminate any potential residue from the disinfectant solution and then subjected to sterilization at 121 °C for 15 min [42,62]. Sil-Ref surfaces were painted with a brush and subjected to sterilization for 30 min using ultraviolet (UV) radiation [37]. Prior to the initiation of biofilm formation, the initial weight of each coupon was aseptically determined.

Cyanobacterial biofilms were developed on agitated 12-well microtiter plates (VWR International, Carnaxide, Portugal) incubated at 25 °C using a 25 mm orbital diameter incubator (Agitorb 200ICP, Norconcessus, Ermesinde, Portugal) at 185 rpm [42]. This setup emulates the hydrodynamic conditions found in marine environments [63], achieving an average shear rate value of 40 s^−1^ [42]. The biofilm formation within this system has also demonstrated its ability to replicate the observed biofouling behavior during prolonged immersion in the sea [64]. Transparent double-sided adhesive tape was then placed in the wells to fix the coupons, following which all the plates were subjected to UV sterilization, and the sterile coupons were fixed on the wells.

Cyanobacterial suspensions from both strains were adjusted to a chlorophyll *a* concentration of 1.11 ± 0.27 µg/mL since the chlorophyll *a* concentration is often used to estimate the biomass of these organisms [42,65]. The cyanobacterial cells were harvested by centrifugation (3202× *g*, for 5 min at room temperature), and a volume of 2 mL of 99.8% methanol (Methanol ACS Basic, Scharlau Basic, Barcelona, Spain) was added. The cyanobacterial suspensions were then incubated for 24 h at 4 °C in the dark, the samples were centrifuged again at 3202× *g*, 5 min at room temperature, and the supernatant was transferred to a glass cuvette. Absorbance measurements were performed at three wavelengths: 750 nm (turbidity), 665 nm (chlorophyll *a*), and 652 nm (chlorophyll *b*) using a V-1200 spectrophotometer (VWR International China Co., Ltd., Shanghai, China). The values were used to determine the chlorophyll *a* concentration (µg/mL) using Equation (1) [66]:

(1) $$Chl\;a\;(µg/mL)=16.29\times A^{665}-8.54\times A^{652}$$

where *A*^665^ and *A*^652^ are the absorbances at 665 nm and 652 nm, respectively. These measurements were obtained in triplicate. Dilutions were performed in Z8 medium with 25 g/L of synthetic sea salts and vitamin B_12_. A volume of 3 mL of cyanobacterial suspension was inoculated per well. Microtiter plates were maintained with a light cycle of 14 h (8–10 μmol photons m^−2^ s^−1^) followed by 10 h of darkness [42]. The biofilm formation was monitored for seven weeks (49 days), as it has been reported that a two-month interval for maintenance is the minimum time for economically viable submerged monitoring systems [42]. The medium was changed twice a week during this period.

### 4.3. Biofilm Analysis

Every 7 days, biofilm analysis was performed, where two coupons of each surface and of each cyanobacterial strain were analyzed. The culture medium was replaced with 3 mL of sterile sodium chloride solution (8.5 g/L) to perform a washing step, thus eliminating loosely attached cyanobacteria [42]. Successively, the wells were filled again with 3 mL of sterile sodium chloride solution to evaluate the architecture of the cyanobacterial biofilms by OCT. The determination of the biofilm wet weight was also performed to complement their characterization.

OCT images were captured and analyzed as previously reported [36,42]. For each coupon, 3D imaging was performed with a minimum of two fields of view to confirm the accuracy of the results. An assessment of the different biofilm structural parameters, such as the biofilm thickness, biovolume, average size of the non-connected pores, and contour coefficient was performed as described in detail by Romeu et al. [36]. Overall, a summary of the parameter definitions and equations is presented in Table 1.

For the determination of the biofilm wet weight, after the analysis from OCT, the sodium chloride solution was removed from the wells, the coupons were detached and weighed, and the biofilm wet weight was obtained as the difference from the initial coupon weight assessed before inoculation.

### 4.4. H_2_O_2_ Disinfection Treatment

After seven weeks of biofilm development, the cyanobacterial biofilms were subjected to disinfection treatment with hydrogen peroxide 30% (H_2_O_2_, Perhydrol^®^, Merck KGaA, Darmstadt, Germany). An H_2_O_2_ solution at a final concentration of 100 mg/L was aseptically and freshly prepared, and dilution was performed in sterile distilled water. The H_2_O_2_ concentration of the stock and work solutions was verified by spectrophotometric determination using potassium titanium (IV) oxalate [67]. A solution of 100 mg/L of H_2_O_2_ was added to each well for 4 h [21,47]. Positive controls were performed by adding sterile distilled water instead of 100 mg/L of H_2_O_2_ solution to the biofilms. A concentration of 100 mg/L was applied since most studies addressing the use of H_2_O_2_ in aquatic and/or marine environments used a range between 1 and 250 mg/L of H_2_O_2_ to evaluate their effects on organisms [8,44,52,68,69].

After 4 h of exposure to H_2_O_2_, the disinfectant solution was discarded and the remaining solution was inactivated for 15 min by a widely used universal neutralizer composed of 30 g/L of polysorbate 80 (VWR Chemicals, Le Havre, France), 30 g/L of saponin (VWR Chemicals, Leuven, Belgium), 1 g/L of L-histidine (Merck, Tokyo, Japan), 3 g/L of lecithin (Alfa Aesar, Karlsruhe, Germany), and 5 g/L of sodium thiosulphate (Labkem, Barcelona, Spain) in 0.0025 M phosphate buffer [70]. Controls to evaluate the adverse effect of the neutralizer on the cyanobacterial biofilms were performed and no effects were observed. To remove the residues of the neutralizer solution, the biofilms were washed with sterile distilled water, and the wells were filled again with 3 mL of sterile sodium chloride solution to evaluate the architecture by OCT and quantify their wet weight after chemical treatment.

### 4.5. Regrowth after the H_2_O_2_ Disinfection Treatment

To evaluate the effect of chemical disinfection on long-term biofilm development, additional coupons for both cyanobacterial strains and the surfaces were used after H_2_O_2_ disinfection. Briefly, after H_2_O_2_ application for 4 h, neutralizer application, and a washing step, these wells were filled again with Z8 medium supplemented with 25 g/L of synthetic sea salts and vitamin B_12_, and the microtiter plates were incubated again in the incubator at the same conditions of biofilm development (25 °C, 185 rpm) for 2 additional weeks. During this period, the medium was also replaced two times a week. The biofilms not previously subjected to H_2_O_2_ disinfection were also used in this regrowth for two weeks (control biofilms). After these two weeks (a total of 9 weeks of biofilm development), the culture medium was removed, the wells were filled with 3 mL of sterile sodium chloride solution (8.5 g/L) to perform a washing step to remove weakly attached bacteria, and, as previously described, the wells were filled again with 3 mL of sterile sodium chloride solution. The structure of the cyanobacterial biofilms developed after two weeks of chemical disinfection, and the respective controls were then analyzed by OCT, and their wet weight was also determined.

### 4.6. Statistical Analysis

Four replicates (two technical replicates in two independent biological assays) were analyzed. The statistical program GraphPad Prism^®^ for Windows, version 6.01 (GraphPad Software, Inc., San Diego, CA, USA) was used for data analysis. The D’Agostino–Pearson and Shapiro–Wilk normality tests were performed to check for normal distribution. Since the variables were not normally distributed, the results of the biofilm wet weight, biofilm thickness, biovolume, average size of the non-connected pores, and contour coefficient were compared using the unpaired, non-parametric Mann–Whitney test. The error bars shown in the graphs correspond to the standard deviation (SD) of the mean. Statistically significant differences were considered for *p* values ≤ 0.1 and the asterisk (*) denotes significance where *p* ≤ 0.1.

## Figures and Tables

**Table 1:** Variables and parameters definition for 3D OCT analysis.

Symbol/Parameter	Description	Formula
*x*	Pixel position on the horizontal axis (width)	*n.a.*
*y*	Pixel position on the vertical axis (height)	
*z*	Pixel position on perpendicular axis (depth)	
*i*	Index of *x*,*z* position in the horizontal plane	
*N*	Number of voxels in the horizontal plane of the Region of Interest (ROI)	
$V_{vox}$	Volume of a voxel (µm^3^)	
$L_{F,i}$	Biofilm thickness at a given position *i* (µm)	
${\bar{L}}_{F}$	Average biofilm thickness (µm)	
$A_{ROI}$	Total area of the ROI (mm^2^)	
${Acon}_{x,z}$	Number of connected voxels identified as belonging to biofilm matrix/bacteria in the biofilm (biovolume) in a horizontal plane at position *y*	
$C_{F,i}$	Number of voxels identified as belonging to biofilm matrix/bacteria in the biofilm (biovolume) in column *i* (vertical line of voxels at position *i*) and connected with the environment (including corner voxels)	
Biovolume	Number of all connected voxels in all images of a horizontal plane multiplied by the voxel size, and it provides an estimate of the biomass in the biofilm (µm^3^) per area of the ROI	$Biovolume\;\left(\frac{{µm}^{3}}{{mm}^{2}}\right)=\frac{\sum_{all\;planes}\left[{(Acon}_{x,z})\times V_{vox}\right]}{A_{ROI}\;}$
Average size of non-connected pores	The determination of the average size of non-connected pores inside the biofilm structure was quantified, assuming the minimum size of non-connected pores equal to 1000 µm^3^ and defining 1 voxel size as corresponding to 117 µm^3^	*n.a.*
Contour coefficient	Number of voxels connected to the background divided by the number of voxels of a horizontal plane	$Contour\;coefficient=\frac{1}{N}\sum_{i=1}^{N}\frac{C_{F,i}}{A_{x,z}}$

## Data Availability

Not applicable.
